# Drug Reaction with Eosinophilia and Systemic Symptoms (DRESS): An Interplay among Drugs, Viruses, and Immune System

**DOI:** 10.3390/ijms18061243

**Published:** 2017-06-09

**Authors:** Yung-Tsu Cho, Che-Wen Yang, Chia-Yu Chu

**Affiliations:** Department of Dermatology, National Taiwan University Hospital and National Taiwan University College of Medicine, Taipei 10002, Taiwan; yungtsucho@gmail.com (Y.-T.C.); emilyyang0123@gmail.com (C.-W.Y.)

**Keywords:** drug reaction with eosinophilia and systemic symptoms, drug-induced hypersensitivity syndrome, histopathology, pathogenesis, human herpesviruses-6, treatment, prognosis

## Abstract

Drug reaction with eosinophilia and systemic symptoms (DRESS) syndrome is a severe multiorgan hypersensitivity reaction mostly caused by a limited number of eliciting drugs in patients with a genetic predisposition. Patients with DRESS syndrome present with characteristic but variable clinical and pathological features. Reactivation of human herpesviruses (HHV), especially HHV-6, is the hallmark of the disease. Anti-viral immune responses intertwined with drug hypersensitivity make the disease more complicated and protracted. In recent years, emerging studies have outlined the disease more clearly, though several important questions remain unresolved. In this review, we provide an overview of DRESS syndrome, including clinical presentations, histopathological features, pathomechanisms, and treatments.

## 1. Introduction

Drug reaction with eosinophilia and systemic symptoms (DRESS) syndrome, which is also termed drug-induced hypersensitivity syndrome (DiHS) by Japanese experts, is one of the drug-induced severe cutaneous adverse reactions (SCARs) [1]. It is a life-threatening disease with cutaneous presentation and internal organ involvement, and its mortality rate is about 10% [2]. The incidence of DRESS is still unclear, with an estimated overall population risk of between 1 in 1000 and 1 in 10,000 drug exposures [3,4]. However, in clinical practice, a diagnosis of DRESS syndrome is frequently overlooked and easily missed due to its variable and complicated manifestations. Greater familiarity with the clinical features and pathogenesis of DRESS syndrome is therefore important for ensuring its correct diagnosis and providing prompt treatment.

In historic terms, the presentation of DRESS syndrome was initially observed in patients receiving anticonvulsants in the 1930s [5]. Subsequently, a case of fever, exfoliative dermatitis, and hepatitis after taking phenytoin was reported in 1950 and termed Dilantin hypersensitivity [6]. In the following years, many different terms were used to describe this clinical syndrome, with those terms primarily depending on the culprit drug, such as allopurinol hypersensitivity syndrome, sulfone syndrome, and anti-convulsant hypersensitivity syndrome. To reduce the confusion resulting from these various terms and to clarify the disease entity, Bocquet et al. [7] proposed the term DRESS, along with a concise description of the syndrome, in 1996. On the other hand, reactivation of human herpesvirus (HHV)-6 was identified in patients with this hypersensitivity syndrome in 1997 [8], followed by 2 other reports from Japan in 1998 [9,10]. Since then, studies and reports regarding DRESS, or DiHS, have placed an emphasis not only on drug responses *per se* but also on the associated virus reactivation or anti-viral immunity.

In this review, we try to organize a concise description of DRESS syndrome and also to address new findings regarding the syndrome in recent years. This review covers many different aspects of DRESS syndrome, including clinical features, histopathological findings, pathomechanisms, and treatments, as well as unresolved problems.

## 2. Clinical Features

The clinical presentations of DRESS syndrome are characterized by fever, widespread skin lesions, internal organ involvement, a long latent period after intake of the inciting drug, a prolonged and protracted clinical course, and possible sequential reactivation of various HHVs [11].

### 2.1. Cutaneous Manifestations

**Figure 1:**
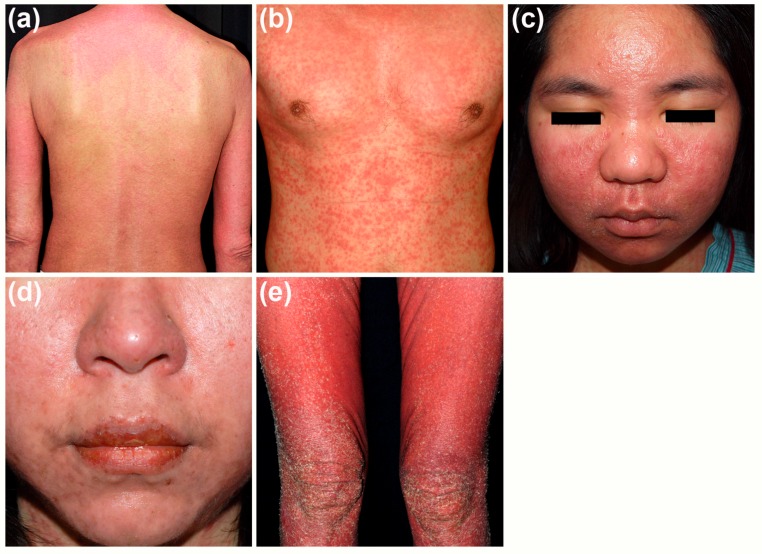
Cutaneous presentations in patients with drug reaction with eosinophilia and systemic symptoms (DRESS) syndrome. (**a**) Widespread purpuric papules and plaques on the trunk and limbs; (**b**) Infiltrative lesions on the trunk; (**c**) Facial edema with peri-orbital sparing; (**d**) Erosions on the lips; (**e**) Desquamation in the stage of resolution.

Skin lesions are most prevalent but not universal presentations in DRESS syndrome. They present in 73–100% of the patients [12]. Skin lesions in patients with DRESS syndrome are generally not specific but are of some common features (Figure 1) [13]. Typically, skin rash involves more than half of the body surface area and may even develop into erythroderma. The lesions are usually infiltrative papules and plaques with markedly purpuric change. These cutaneous lesions are frequently of polymorphic presentations, which can be reported as maculopapular, urticarial, exfoliative, lichenoid, pustular, bullous, target-like, or eczema-like lesions. Facial edema, which can be found in 76% of patients, is the hallmark feature of the disease [14]. Later, desquamation presents in the stage of resolution.

The infiltrative and purpuric skin rash could correspond to infiltrations of inflammatory cells in skin lesions and evidence of vessel wall damage in histopathological findings of DRESS syndrome (discussed later in Section 3). The presence of facial edema is a characteristic presentation in DRESS syndrome, one which might be a predictor of a more serious reaction than drug-induced maculopapular exanthema (MPE) [15]. The mechanism underlying the development of facial edema is still unknown, but it may be related to the involvement of the vascular endothelial growth factor pathway [16]. Mucosal lesions are frequently reported and can be found in more than 50% of DRESS cases, with the mouth and lips being the most commonly affected area [14,17].

### 2.2. Internal Organ Involvement

Both hematological abnormalities and impairments of solid organs may be observed in patients with DRESS syndrome. In terms of hematological changes, eosinophilia is the most common one, being present in 66–95% of patients [11,14]. In addition to eosinophilia in the blood, tissue infiltrations of eosinophils are also evident and could be related to the damage caused to these organs [18]. Similar with eosinophils, atypical lymphocytes are present in both blood and tissues. Upon examination, atypical lymphocytosis in the blood can be identified in 27–67% of patients [2,11,14]. In addition, lymphadenopathy can be found in 54% of patients by physical examinations or image studies [14]. Beyond the results of routine examinations, some studies have revealed decreased numbers of B lymphocytes with hypoglobulinemia in patients in the early phase of DRESS syndrome [19,20,21].

Multiple internal organs may be damaged over the course of DRESS syndrome. Liver injury is the most common type of organ damage and has been found in 75–94% of patients [2,11,14]. Such damage may occur before the onset of cutaneous lesions and may be associated with the presence of atypical lymphocytes in the blood [22]. Compared to those seen in other SCARs, the liver injury seen in DRESS syndrome tends to be more severe and to last longer [23]. According to the International Consensus Meeting Criteria, the different types of liver injury can be further divided into three patterns based on the values of alanine aminotransferase (ALT) and alkaline phosphatase (ALP) at the initial presentation, with the three types being termed the cholestatic type, hepatocellular type, and mixed type [24]. In brief, the determination of the type of liver injury is based on the value of R ratio, which is the ratio of serum ALT results to ALP results with respect to their upper limits of normal range (ULNs) [*R* = (serum ALT/ULNs of ALT)/(serum ALP/ULNs of ALP)]. Liver injury is defined as cholestatic type when R ratio less than 2, hepatocellular type when R ratio greater than 5, and mixed type when R ratio greater than 2 but less than 5 [24]. Among DRESS patients with liver injury, cholestatic type is the most prevalent one and can be found in 44% of the patients, followed by mixed type (33%) and then hepatocellular type (23%) [22]. In addition, cholecystic type liver injury is linked to older patient age and a higher frequency of interface change in skin biopsy specimens [22]. At the same time, it should be noted that the patterns and severity of liver injury are not related to either the culprit drugs or the treatments for DRESS syndrome [22].

Renal involvement is also prevalent in patients with DRESS syndrome, occurring in around 12–40% of patients [2,11,14]. Risk factors for the development of drug-induced kidney injury include older age and underlying renal or cardiovascular diseases [25]. Among the culprit drugs associated with renal involvement, allopurinol is the most notorious one [26]. Allopurinol-induced SCAR is significantly associated with the human leukocyte antigen (HLA)-B*58:01 allele in Han Chinese patients with a gene dosage effect [27,28]. Allopurinol-induced SCAR is more likely to develop in patients with underlying renal impairment because of the prolonged clearance of an allopurinol metabolite, oxypurinol, observed in these patients [29,30]. Renal involvement in patients with DRESS syndrome is usually mild and will be recovered from in time without obvious sequelae. However, in some cases, severe interstitial nephritis, acute tubular necrosis, or vasculitis could develop and may lead to renal failure or even mortality [31]. Short-term or long-term hemodialysis may be needed in such cases [2,32].

Lung involvement is the third most common type of organ involvement seen in DRESS syndrome, occurring in about one-third of DRESS patients [2,14]. Pulmonary involvement may present with impaired pulmonary function, interstitial pneumonitis, pleuritis, and acute respiratory distress syndrome [18,31]. Such lung involvement has been linked to the usage of minocycline [26]. Most of the cases with lung involvement recover smoothly, with the exception of a few cases presenting with acute respiratory distress syndrome in which mechanical ventilation is required [18,31]. In such cases, minocycline and acabavir are the main culprit drugs leading to severe pulmonary involvement [31].

Cardiac involvement has been reported in 4–27% of the patients in DRESS syndrome [33] and might be highly fatal when it occurs [34]. Patients with cardiac involvement usually present with left ventricular dysfunction and electrocardiographic changes [35]. Some medications are more frequently reported to be linked to cardiac involvement, including minocycline, ampicillin, and sulfonamides [34,35]. Such patients may exhibit chest pain, dyspnea, tachycardia, and hypotension. Cardiac involvement includes two forms of reactions: hypersensitivity myocarditis and acute necrotizing eosinophilic myocarditis. Hypersensitivity myocarditis is usually mild and self-limited after withdrawal of the inciting drugs, thus causing an under-recognized rate of cardiac involvement. Acute necrotizing eosinophilic myocarditis is a more severe form of hypersensitivity and results in rapid deterioration with a high mortality rate of more than 50% [35]. Prompt identification with adequate treatment is mandatory for patients with cardiac involvement.

The neurologic manifestations of DRESS syndrome include headache, seizure, coma, and motor function impairment. These may result from meningitis or encephalitis [18,36]. The involvement of other organs is occasionally encountered, including the pancreas, gastrointestinal tract, and spleen [11,14,18].

### 2.3. Culprit Drugs

Many drugs have been reported to be a causative agent of DRESS syndrome. However, only a limited number of drugs are frequently encountered as culprits (Table 1), including anti-convulsants, anti-microbial agents, anti-viral agents, antipyretic agents, and others. The most peculiar feature of these culprits is a long latent period, which ranges from 3 to 8 weeks after commencement of the drugs (see Section 2.4. clinical courses).

In addition, several newly developed drugs have also been reported as DRESS syndrome culprits. These include anti-hepatitis C virus agents (boceprevir and telaprevir) [42,43,44], targeted therapies for oncological diseases (sorafenib [46], vismodegib [47], and vemurafenib [48]), a new anti-coagulant (rivaroxaban) [49], and a new uric acid-lowering agent (fubuxostat) [50]. Although these agents are not notorious for inducing DRESS syndrome, such reports reflect the fact that with increasing introductions of new drugs, the list of culprit drugs for DRESS syndrome will continue to grow.

### 2.4. Clinical Courses

**Figure 2:**
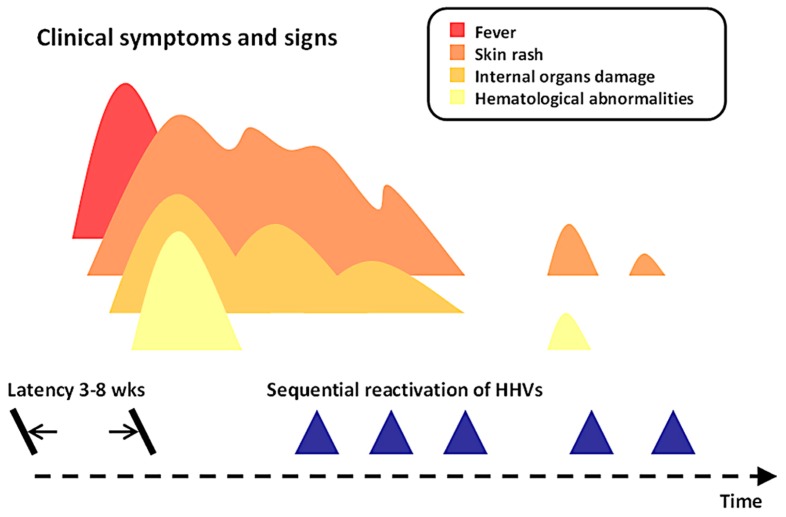
Clinical courses of patients with DRESS syndrome.

The most characteristic feature of the clinical course of DRESS syndrome is a delayed onset and a prolonged and protracted evolution of the disease (Figure 2). Patients with DRESS syndrome typically develop symptoms and signs of drug reaction at least 3 weeks after the start of the eliciting drug. The latency period of DRESS syndrome is longer than those of Stevens-Johnson syndrome (SJS), toxic epidermal necrolysis (TEN), acute generalized exanthematous pustulosis, fixed drug eruptions, and MPE, which all belong to delayed type hypersensitivity [11,14,18]. This longer period of latency is a special feature of DRESS syndrome and may result in a failure to properly make the diagnosis.

At the beginning, patients may experience some prodromal symptoms before or along with the development of skin rash. These symptoms include fever, pruritus, dysphagia, pain, or lymph node enlargement [14,51]. Internal organ damage and hematological abnormalities can also be detected at that time or at a later period. It is difficult, in fact, to know the exact time point at which the development of organ damage and blood alterations occur except in the cases of already hospitalized patients. One recent report showed that liver injury in DRESS syndrome may develop before the onset of skin eruptions in 9.7% of patients [22].

The duration of illness of DRESS syndrome is usually more than 15 days, with a waxing-and-waning quality in which several flare-ups may occur after recovery from the initial presentation. In a study by Tetart et al., 7 of the 32 patients (22%) still had symptoms and signs 3 months after the disease onset, with symptoms lasting for more than half a year in 4 cases (13%) and even up to 1 year in 3 cases (9%) [52]. Risk factors for a prolonged evolution of the clinical course in DRESS syndrome may include a higher level of baseline lymphocytosis, a higher value of liver enzyme levels, ethnicity, and culprit drugs [52]. In past studies, the protracted clinical courses of DRESS syndrome have been linked to the reactivations of several herpesviruses [53,54,55,56]. These reactivations may follow an order resembling those that occur in patients with graft-versus-host disease [56]. First, it would be Epstein-Barr virus (EBV) and/or HHV-6, followed by HHV-7 and later cytomegalovirus (CMV). Recent reports showed that reactivations of these herpesviruses could also develop in patients with other SCARs, including SJS/TEN and MPE [57,58]. However, it is noteworthy that the reactivation of HHV-6 was found almost exclusively in patients with DRESS, or DiHS, with a frequency of 43–100% in such patients [57,58]. This could lead to an assumption that the role of HHV-6 is important and unique in DRESS syndrome.

On the other hand, Picard et al. reported that 15 of the 60 patients (25%) in their study had recurrences of the disease after using a structurally unrelated new drug within an unusually short period of time, with more than 50% of those cases having more than one episode [59]. The severity of these recurrences of the disease was mild in most of the cases and the presentations were usually incomplete. Skin eruptions were the most prevalent presentation of the recurrences, followed by eosinophilia in about half of the cases and internal organ damage in 13% of the cases [59]. The exact pathomechanism of these recurrences are still unknown. Cross-reactivity is one of the possible explanations for this phenomenon but is largely debated due to the situation of which molecular mimicry does not exist [59]. Neosensitization is one plausible mechanism with evidences from in vivo tests to both original and new structure-unrelated drugs [60]. Another possible explanation is the reactivation of human herpes virus with subsequent immune alteration caused by the new drug introduction. This is evident from the in vitro observation that amoxicillin could induce HHV-6 replication in a human T lymphoblastoid cell line [61]. As abovementioned, viral reactivation has been linked to the protracted and fluctuated clinical courses of DRESS syndrome. However, to better explore the underlying mechanism, further studies to identify and compare the compositions and clonalities of activated T cells during the acute and the recurrent episodes may provide more details of information.

### 2.5. Diagnostic Criteria

The diagnosis of DRESS syndrome requires a vigilant mind, careful clinical observations, and a thorough laboratory examination. Multiple differential diagnoses may mimic DRESS syndrome with a very familiar presentation. These include infectious mononucleosis, anti-retroviral syndrome, systemic lupus erythematosus, and so on [62].

The most used diagnostic criteria are included in the scoring system proposed by the RegiSCAR group (Table 2) [13,17]. This scoring system comprises the major features of DRESS syndrome, giving each item a score of minus one point, zero points, one point, or two points. The diagnosis of DRESS syndrome is then made based on the total score: <2 points: no case; 2–3 points: possible case; 4–5 points: probable case; >5 points: definite case.

Another commonly used standard is the diagnostic criteria for DiHS, which were proposed by a Japanese consensus group [63]. The criteria comprise 7 items, which are quite similar to those in the RegiSCAR criteria. The most important difference is that HHV-6 reactivation is included in the diagnostic criteria for DiHS. This difference does not, however, go against the notion that DRESS syndrome and DiHS are parts of a continuum of the same disease. In fact, patients with typical DiHS may represent a severe form of DRESS syndrome [64].

### 2.6. Prognosis and Long-Term Sequelae

DRESS syndrome is a life-threatening disease with a mortality rate of around 10% [2]. Patients may also suffer morbidities due to DRESS syndrome-related organ damage or due to treatment-related complications. The damage to organs can be very severe in patients with DRESS syndrome, leading to permanent functional impairment of the affected internal organs. Liver transplantations have been used in patients with severe liver damage [18]. Patients with underlying chronic kidney disease are prone to having marked and permanent deterioration of kidney function and may require lifelong hemodialysis. Infections are among the major complications due to treatment for DRESS syndrome, including herpes labialis, herpes zoster, pneumonia, and soft tissue abscess [65]. The severity of such infections can be quite severe, potentially even leading to septic shock or death [2]. These infections are more likely to develop in patients receiving systemic corticosteroids than in those receiving supportive care [65]. Fulminant type 1 diabetes mellitus (DM) was also reported to have developed in 5 out of 145 patients with DRESS syndrome in a follow-up study in Asia [66]. It usually occurs 1–2 months after the resolution of DRESS syndrome. Onuma et al. found a higher frequency of HLA-B*62 in fulminant type 1 DM associated with DiHS than in those without DiHS [67]. Although the mechanism for the development of fulminant type 1 DM in patients who have recovered from DRESS has been reported to be associated with HHV-6 reactivation, the exact mechanism is still largely unknown.

In addition, thyroid diseases are the most frequently encountered long-term sequelae in DRESS syndrome patients, with a reported rate of 4.8% [66,68]. Thyroid diseases include Graves’ disease, Hashimoto’s thyroiditis, and painless thyroiditis. Of note, in one previous study, anti-thyroglobulin or anti-thyroperoxidase antibodies could be detected in 7 out of 16 patients who had recovered from DiHS without clinical presentations of thyroiditis [65]. This implies that the actual rate of development of autoimmune thyroid diseases might be even higher than that previously reported. A possible link of HHV-6 reactivation to the development of these thyroid diseases has been proposed because of a higher rate of detection of HHV-6 in the thyroids of patients with Hashimoto’s thyroiditis than in the thyroids of controls [69]. In fact, HHV-6 has been shown to play a role in the development or triggering of Hashimoto’s thyroiditis even in patients without DRESS syndrome [70].

Other than thyroid diseases, several autoimmune diseases have also been reported in patients who have recovered from DRESS, including systemic lupus erythematosus [71], autoimmune hemolytic anemia [68], reactive arthritis [72], alopecia areata [68], and vitiligo [66]. These autoimmune sequelae develop after the resolution of DRESS syndrome over a wide range of time, from a few months to several years [66]. The exact mechanism for the development of these autoimmune diseases is still unknown, but it has been postulated that they develop due to the dysfunction of regulatory T cells (Tregs) in the resolution phase of DRESS syndrome [66,68]. In accordance with this assumption, an observation of the presence of anti-plakin autoantibodies in about 60% of the patients in the late resolution phase (>100 days from the index date) of DiHS has been reported [73].

## 3. Histopathology

**Figure 3:**
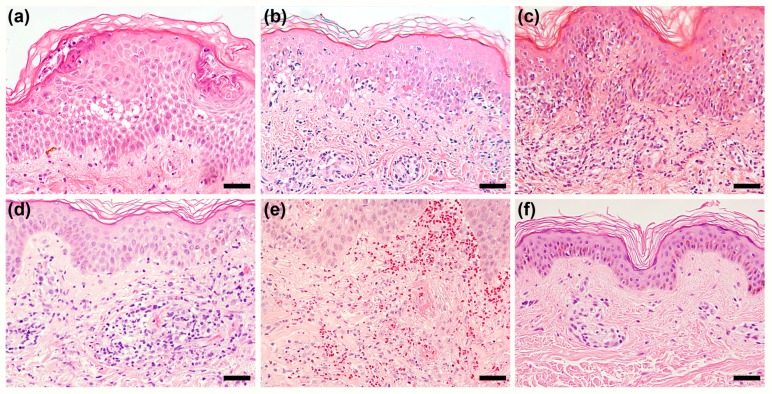
Histopathological patterns in skin lesions of patients with DRESS syndrome. (**a**) Spongiosis with focal subcorneal pustules; (**b**) Erythema multiforme-like interface dermatitis: basal vacuolar change and multiple scattered apoptotic keratinocytes in the epidermis; (**c**) Lichenoid dermatitis: prominent infiltrations of cells in the upper dermis and basal vacuolar change; (**d**) Vascular damage: perivascular infiltration with prominent endothelial cells, red blood cell extravasation, and some extent of vessel wall damage, resembling a feature of lymphocytic vasculitis; (**e**) Leukocytoclastic vasculitis: frank fibrinoid necrosis, leukocytoclasia, and red blood cell extravasation; and (**f**) Superficial perivascular dermatitis. All panels were prepared using H&E stain, with a magnification of 200×. Scale bar = 50 μm

The histopathological features of patients with DRESS syndrome are generally non-specific. There is no single unique finding that can be used to differentiate DRESS syndrome from other drug eruptions or inflammatory skin disorders. In past years, several commonly encountered histopathological patterns have been identified in the skin specimens of patients with DRESS syndrome (Figure 3), including spongiosis, interface dermatitis, vascular damage, and superficial perivascular infiltration [74].

Interface dermatitis is the most common histopathological presentation, having been found in more than three-fourths of the patients with DRESS syndrome in most previous studies [74,75,76]. It consists of an erythema multiforme-like pattern and/or a lichenoid dermatitis pattern. The degree of apoptotic keratinocytes seen in interface dermatitis has been demonstrated to be correlated with the severity of liver injury [77], of renal injury [76], and of the overall disease [78]. We can assume that a higher degree of apoptotic keratinocytes, which are usually caused by CD8^+^ T-cells, indicates a higher degree of cytotoxicity and thus explains these more severe levels of tissue damage.

Spongiosis is also a commonly seen feature of the histopathological presentation, having been found in 40–80% of the cases in previous studies [74,75,76,77]. In one study, the presence of spongiosis was shown to be associated with a milder severity of the disease [78]. In some cases, subcorneal pustules can be seen even though there are no obvious cutaneous pustules in these patients. In addition, the feature of vascular damage is also prevalent in patients with DRESS syndrome, having been observed in 54–88% of the patients in prior studies [74,76,77,78]. The extent of vascular damage seen in those studies varied largely but at least included findings of prominent endothelial cells, red blood cell extravasations, and a certain degree of vessel wall damage [74]. Otherwise, while vascular damage is prevalent in DRESS syndrome patients, frank leukocytoclastic vasculitis was seldom seen in most previous studies excepting one [78]. In that study, Skowron et al. found that 28% of their patients exhibited the feature of leukocytoclastic vasculitis.

Perivascular infiltration, which may comprise lymphocytes, eosinophils, neutrophils, and atypical lymphocytes of various amounts, is a universal feature of all DRESS syndrome cases [74,75,76,77,78]. The intensity of lymphocyte infiltration has been linked to the severity of liver injury and to the degree of blood eosinophilia [79]. In some cases, superficial perivascular infiltration is the only pathological feature to be observed [74,75]. In addition, peri-appendage infiltration could occasionally be found [74,75,79]. The significance of peri-appendage infiltration has not been identified yet, but it might be linked to the reactivation of HHV-6 and possible autoimmune sequelae [79].

Although the histopathological features of patients with DRESS syndrome are not specific, there are still some characteristic findings that might be a clue for diagnosis or an indicator for severity. The most important such finding is the co-existence of the aforementioned patterns in a single skin specimen. Around 50–60% of DRESS syndrome patients have at least 2 of the aforementioned patterns in a single specimen [74,75]. Relatedly, patients with DRESS syndrome tend to have more histopathological findings in a single skin specimen than those with MPE [80]. Moreover, patients with three histopathological patterns (spongiosis, interface dermatitis, and vascular damage) co-existing in a single specimen have a significantly higher likelihood of being a definite case of DRESS syndrome, may have a higher degree of hematological abnormalities, and show a trend toward having a higher rate of HHV-6 reactivation [74].

## 4. Pathomechanisms

**Figure 4:**
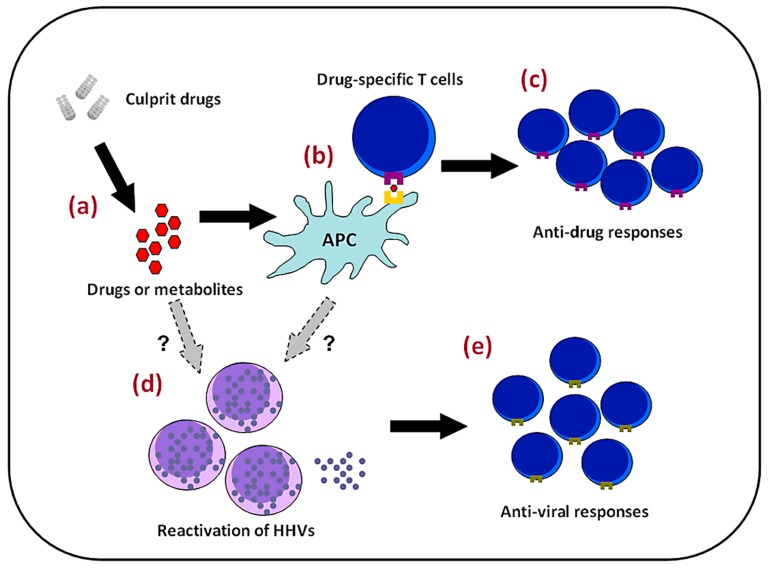
Possible pathomechanisms of DRESS syndrome. Drugs or their metabolites (**a**) may accumulate in some persons due to altered activity of metabolizing enzymes; (**b**) Evoking drug-specific T lymphocytes in persons with certain genetic backgrounds; (**c**) Causing the clinical presentations of DRESS syndrome; On the other hand, (**d**) viral reactivations, which result from a direct effect of the inciting drugs or their metabolites or from a “cytokine storm” caused by anti-drug immune responses; and (**e**) May induce robust anti-viral responses contributing to the development of the disease.

The pathomechenisms of DRESS syndrome are complex and still largely unknown (Figure 4). Trying to resolve this problem is akin to collecting numerous puzzle pieces in order to complete a jigsaw puzzle, and it is clear that some important pieces are still missing. Current evidence shows that DRESS syndrome tends to occur in genetically predisposed persons when they are ingesting one of the aforementioned inciting drugs. In addition to drug hypersensitivity, the reactivation of HHVs and subsequent anti-viral immune responses may also contribute to a higher severity and a more protracted course of DRESS.

### 4.1. Genetic Factors

Emerging evidences show that genetic factors play various roles in different aspects of the pathomechanisms of DRESS syndrome [18,62,81]. One such factor is the polymorphism in genes encoding metabolizing enzymes for drugs, such as cytochrome P (CYP) 450 enzyme and N-acetyltransferase. Reduced activities of these metabolizing enzymes cause the accumulation of drugs or their active metabolites, which can then interact with cellular proteins or peptides, in turn evoking immune responses. One recent report showed that 16 single-nucleotide polymorphisms in *CYP2C* genes are associated with phenytoin-related SCARs [82]. Among these polymorphisms, CYP2C9*3 showed the most significant association, with an overall odds ratio of 11 in an Asian population. In addition, the delayed clearance of phenytoin in the blood was also found in patients with phenytoin-related SCARs, especially those carrying CYP2C9*3. Other than phenytoin-related SCARs, several anticonvulsant-related SCARs were also found to be related to these metabolizing enzymes. Anticonvulsants are converted by the CYP 450 system to arene oxide metabolites, which are then further metabolized by epoxide hydroxylase or glutathione transferase. Previous studies have shown that mutations in epoxide hydroxylase result in the accumulation of toxic metabolites and then elicit hypersensitivity reactions [83,84]. A similar scenario has been found in sulfonamide-related DRESS syndrome. A slow metabolizing capacity in the *CYP450* system results in the accumulation of toxic hydroxylamine metabolites, which may account for a predisposing factor of sulfonamide-related SCARs [85].

Another aspect of the pathomechanisms of DRESS syndrome which could be influenced by genetic factors is the immune response *per se*, especially with respect to the influence of polymorphisms in genes encoding HLA molecules [86]. A number of emerging studies have revealed associations between polymorphisms in HLA alleles and the development of DRESS syndrome (Table 3). HLA alleles determine the structure of the major histocompatibility complex (MHC) and are involved in antigen presentations and in the formation of immunological synapses. Therefore, polymorphisms of HLA alleles influence what kinds of antigens are presented and influence subsequent T lymphocyte responses.

Polymorphisms in HLA alleles largely explain the genetic predisposition of patients with DRESS syndrome. However, some critical problems remain unresolved. In most cases, the HLA alleles in question are also linked to the development of SCARs other than DRESS syndrome. For example, people bearing HLA-B*58:01 have increased risks for the development of both allopurinol-induced DRESS syndrome and SJS/TEN. In addition, HLA-A*31:01 increases the risk of developing carbamazepine-induced DRESS syndrome but also increases the risk of developing SJS/TEN syndrome in individuals of Japanese and European descents [92,93]. Therefore, it is not unreasonable to assume that the repertoire of T cells is the determinant for the development of SCARs. It is evident that allopurinol-specific T cells could be generated from patients with allopurinol-induced SCARs but not from those who tolerate allopurinol, even though all these subjects bear the HLA-B*58:01 allele [99]. However, it is still not known if the types or clones of T cells being activated are the major factor in determining which type of SCAR a given patient develops. To clarify this issue, further studies may focus on the differences among T cell clonalities or responses activated in different SCARs. Of note, for lots of the culprit drugs, a genetic predisposition for patients with DRESS syndrome is still un-identified. Further efforts to find such associations are warranted.

Three non-mutually exclusive models have been proposed to explain the interactions between drugs or metabolites and immunological synapses, namely, the hapten/pro-hapten model, the pharmacologic interaction (p-i) model, and the altered peptide repertoire model [100]. In the hapten/pro-hapten model, drugs or metabolites bind covalently to endogeneous proteins, being processed and presented by antigen-presentation cells, and are recognized as foreign antigens. In the p-i model, it is hypothesized that drugs or metabolites can bind non-covalently to MHC proteins or T-cell receptors (TCRs) in a peptide-independent manner to elicit T cell responses. In the altered peptide repertoire model, drugs and metabolites bind directly to the binding groove of MHC proteins, changing the peptide specificity of MHC binding. Then, these peptides are recognized as foreign and then evoke T cell responses.

The activated T cells in DRESS syndrome are complex. Hansel et al. had reported a case of DRESS syndrome caused by ceftriaxone which had been confirmed by patch testing [101]. Immunohistochemical study of skin biopsies from both the exanthema and the patch test revealed positive results for interleukin (IL)-5, perforin, granzyme B, fatty acid synthase ligand (FasL), and interferon (IFN)-γ, which supported the notion that DRESS syndrome belongs to type IVb/IVc hypersensitivity reaction [101]. In addition, the activated T cells in DRESS syndrome have been shown to exhibit anti-EBV capacities [102]. Expanded CD8^+^ T cells from patients with DRESS syndrome could recognize one of several EBV epitopes and are capable of the production of large amounts of tumor necrosis factor (TNF)-α and IFN-γ. These observations could be explained by the notion of heterologous immunity [100,103]. Heterologous immunity here indicates that drug-specific T cells are derived from the cross reactivity of pathogen-specific effector memory T (T_EM_) cells sensitized much earlier. These T_EM_ cells would become activated after drug exposure and thus result in development of the disease. Among the pathogens being considered, HHVs are thought to be the most likely sources because of their capacities for persistent infection and intermittent reactivation [100]. Several studies have shown that CMV-specific T cells constitute the major proportion of the memory T cell repertoire in patients with latent CMV infection (10–40% of the CD4^+^ T cell repertoire and up to 10% of the CD8^+^ T cell repertoire) and that the number of these CMV-specific T cells increases with age [104,105]. Pathogen-specific T_EM_ repertoires may differ among people who have encountered different pathogens while growing up. Therefore, heterologous immunity might also provide an explanation for why different SCARs develop in patients with the same genetic background in terms of HLA alleles. However, further studies are needed to address these issues and provide more solid evidence to confirm the assumption.

### 4.2. Viral Reactivation

Viral reactivation, especially HHV-6 reactivation, is an important and characteristic feature in patients with DRESS syndrome. Monomyeloid cells are one of the reservoirs of latent HHV-6 infection in humans [106]. One previous study has shown that circulating CD11b^+^CD13^+^CD14^−^ CD16^high^ monomyeloid precursor cells in patients with DRESS syndrome, or DiHS, harboring HHV-6 express a skin homing molecule, C-C motif chemokine receptor (CCR) 4 [107]. These circulating monomyeloid precursors respond to high-mobility group box (HMGB)-1, which has been found in high levels in the skin and blood in patients with DRESS syndrome [108], and then infiltrate into the skin. These skin-infiltrated monomyeloid precursor cells may then transmit HHV-6 to skin resident CD4^+^ T cells [107]. CD4^+^ T cells in the acute phase of DRESS syndrome, or DiHS, have been found to express a higher level of CD134, which is a cellular receptor for HHV-6, than those in SJS and MPE [109]. Therefore, it has been postulated that the skin might be the primary site for initiating HHV-6 reactivation [107].

However, the mechanisms for HHV-6 reactivation are still unknown. Two possible explanations have been proposed. One is a direct effect of drugs or metabolites on viral reactivation. One in vitro study has found, for example, that amoxicillin could induce HHV-6 replication in a human T lymphoblastoid cell line [61]. Similar observations have been made regarding valproic acid, which increases replications of HHV-6 [110] and CMV [111]. Another plausible explanation is the notion of a “cytokine storm”. Previous studies have demonstrated that in the early stage of DRESS syndrome, or DiHS, the number of Treg cells expands [112], even as a reduced number of B cells and hypogammaglobulinemia are evident [19,20,21]. This immunocompromised status has been supported by one recent study, which showed that many pro-inflammatory cytokines and chemokines, such as TNF-α, IFN-γ, IL-1, IL-2, IL-6, are seen in lower levels in the early stage of the disease in patients with HHV-6 reactivation than in those without HHV-6 reactivation, with the exception of one chemokine, interferon γ-induced protein (IP)-10 [58]. IP-10, also known as C-X-C motif chemokine (CXCL) 10, attracts cells bearing C-X-C motif chemokine receptor (CXCR) 3 molecules. The number of plasmacytoid dendritic cells (pDCs) in patients with DRESS, or DiHS, has been shown to be decreased in the blood and increased in the skin around the time of viral reactivation [113]. Plasmacytoid dendritic cells harbor CXCR3 molecules, could release large amounts of type 1 interferon upon activation, and have a prominent anti-viral capacity [114]. This reduced number of pDCs in circulation has been thought to be related to the reactivation of viruses [113]. In addition, serum thymus and activation-regulated chemokine (TARC) levels are markedly elevated in the acute stage of DRESS syndrome, or DiHS [115,116]. The levels of TARC are even significantly higher in those with HHV-6 reactivation than in those without HHV-6 reactivation [115]. TARC is a ligand for CCR4 [117] and plays an important role in T helper type 2 (Th2) immune responses [118]. However, whether TARC itself is involved in the recruitment of monomyeloid precursor cells or whether a Th2 immune milieu further dampens anti-viral immune response is still unknown and requires further investigation.

The dysfunction of Treg cells in the resolution phase of DRESS syndrome, or DiHS, has been demonstrated in previous studies, and may account for the sequential reactivation of HHVs and for the development of autoimmune sequelae. This observation has also been supported in another recent report [119]. Niu et al. used TCR repertoire analysis to evaluate the dynamic reformation of the T cell repertoire hierarchy in patients with DRESS syndrome. They found that the extent of fluctuation of CD8^+^ T cell clones correlated positively with clinical severity. In addition, anti-herpesvirus responses and the proportion of the homologous CD8^+^ EBV-specific T cell clonetypes were higher in patients with more fluctuant repertoires. The authors propose that this observation supports the notion that herpesvirus-mediated continuous de novo priming of newly pathogenic CD8^+^ T cell clones plays a role in the pathogenesis of DRESS syndrome [119].

Of note, as aforementioned, reactivation of HHV-6 could be found in 43–100% of the patients with DRESS or DiHS. That is, reactivation of HHV-6 is not an essential event to develop the disease but might be an aggravating factor resulting in a protracted and fluctuated course. Further studies should emphasize on the differences between DRESS patients with and without HHV-6 reactivation and on identifying the exact mechanisms of viral reactivation.

## 5. Treatments

Immediate withdrawal of the inciting drugs is the most important action to take in the management of patients with DRESS syndrome. Most systemic treatments for patients with DRESS syndrome, meanwhile, lack sufficient clinical evidence, as most of them have been derived from case series or experts’ opinions [18,62]. By far, systemic corticosteroids are the mainstay treatment for patients with DRESS syndrome. A starting dose of prednisolone or an equivalent of 0.5–1.0 mg/kg/day with a gradual tapering over 2–3 months has been suggested [18,62]. This gradual tapering over a longer duration may reduce the likelihood of disease flare-ups and reduce the development of autoimmune long-term sequelae [18,62]. However, systemic corticosteroids may be associated with a higher rate of opportunistic infections and with the possibility of lots of complications. Thus, individual adjustments are needed for each case based on the severity of the disease and underlying co-morbidities.

Supportive care only may also be an option in the treatment of patients with DRESS syndrome. Uhara et al. reported on 12 patients with DiHS who received hydration with or without topical steroids [120]. All of these 12 patients recovered well, without complications of infections, within 7 to 37 days after the withdrawal of the inciting drugs. A case series reported by Ushigome et al. also included 17 cases of DiHS treated with only supportive care [65]. All of these cases recovered smoothly without significant complications except for a higher rate of detectable autoantibodies and a higher rate of autoimmune sequelae after recovery from the disease. In addition, Funck-Brentano et al. reported a retrospective study including 50 consecutive patients with DRESS syndrome [121]. Among them, 38 patients with probable or definite DRESS syndrome were further divided into a topical steroid group and a systemic steroid group. Systemic steroids were only used in those patients who presented with at least one life-threatening organ failure. The researchers found higher rates of infections, septicemia, and the need for intensive care in patients in the systemic steroid group, and concluded, furthermore, that systemic steroids may not be required for the treatment of mild forms of DRESS syndrome and should be reserved for those with severe presentations. Nevertheless, there is still lack of a consensus to what degree of severity should systemic corticosteroids commence. One group of the French society of Dermatology has recommended to use systemic corticosteroids when presence of 5-folds elevation of serum transaminase levels, or involvement of either other organs, such as kidney, lung, and heart [122].

Intravenous immunoglobulin (IVIG) is another treatment option that has yielded conflicting results. Several past studies have reported the successful treatment of DRESS cases with IVIG [123,124,125]. However, in one recent study, Poly et al. reported on 6 DRESS syndrome patients who received IVIG [126]. Among them, 5 of the patients suffered severe adverse effects, with 4 patients requiring systemic corticosteroids due to the adverse effects of IVIG or uncontrolled diseases. Thus, the authors suggested that IVIG should not be used as monotherapy in treating DRESS syndrome. Otherwise, several immunosuppressive agents other than corticosteroids have been reported to exhibit treatment effectiveness, including cyclosporine [127], cyclophosphamide [128], mycophenolate mofetil, and rituximab [123]. However, due to only a limited number of patients having received these agents, further studies are required to examine the benefits of these immunosuppressants. In addition, anti-viral treatment such as ganciclovir has been proposed by some doctors in addition to systemic corticosteroids or IVIg to be used in patients with severe disease with confirmation of viral reactivation [122]. However, such management is not validated by well-designed controlled studies and should be thoroughly considered by the judgment between benefits and harms.

## 6. Conclusions

DRESS syndrome is a complex disease that is comprised of complicated interactions among drugs, viruses, and immune responses. As a result of past research endeavors, the understanding of DRESS syndrome has gradually increased. However, some major issues still require clarification, such as what determines the development of different types of CD8^+^ drug-specific T cells in individuals of the same genetic background in terms of HLA alleles, the mechanisms of immunosuppression in the early phase of the disease, and the key steps of HHV reactivation. Further investigations are warranted to address these issues and may shed light on the prevention of or new treatments for the disease.

## Figures and Tables

**Table 1:** The common culprit drugs in drug reaction with eosinophilia and systemic symptoms (DRESS) syndrome.

Category	Drugs
Anti-convulsants	Carbamazepine [2,11,14,18], lamotrigine [2,11,14,18], phenobarbital [11,14,18], phenytoin [2,11,14,18], oxcarbazepine [11,14], gabapentin [37]
Anti-bacterial	Amoxicillin [11,14], ampicillin [14,18], azithromycin [38], levofloxacin [39], minocycline [11,14,18], piperacillin/tazobactam [40], vancomycin [11,14,18]
Anti-tuberculosis	Ethambutol [18,41], isoniazid [2,18,41], pyrazinamide [18,41], rifampin [18,41], streptomycin [11,18,41]
Anti-retroviral agents	Abacavir [11,18], nevirapine [11,14,18]
Anti-hepatitis C virus agents	Boceprevir [42,43], telaprevir [42,44]
Anti-pyretic/analgesics	Acetaminophen [45], diclofenac [2], celecoxib [11,18], ibuprofen [11,18]
Sulfonamides	Dapsone [2,11,14,18], sulfamethoxazole-trimethoprim [2,11,14,18], sulfasalazine [2,11,14,18]
Targeted therapeutic agents	Dorafenib [46], vismodegib [47], vemurafenib [48]
Others	Allopurinol [2,11,14,18], chinese herbal medicine [2], imatinib [11], mexiletine [11,18], omeprazole [11], strontium ranelate [11]

Bold characters highlight the most frequently reported culprit drugs in the literature, which are with a frequency of more than 10 cases ever reported.

**Table 2:** The RegiSCAR scoring system for diagnosing DRESS syndrome.

Items	Score			Comments
	−1	0	1	
Fever ≧ 38.5 °C	N/U	Y		
Enlarged lymph nodes		N/U	Y	>1 cm and ≧ 2 different areas
Eosinophilia ≧ 0.7 × 10^9^/L or ≧ 10% if WBC < 4.0 × 10^9^/L		N/U	Y	Score 2, when ≧ 1.5 × 10^9^/L or ≧ 20% if WBC < 4.0 × 10^9^/L
Atypical lymphocytosis		N/U	Y	
Skin rash Extent > 50% of BSA Rash suggesting DRESS	N	N/U U	Y Y	Rash suggesting DRESS: ≧ 2 symptoms: purpuric lesions (other than legs), infiltration, facial edema, psoriasiform desquamation
Skin biopsy suggesting DRESS	N	Y/U		
Organ involvement		N	Y	Score 1 for each organ involvement, maximal score: 2
Rash resolution ≧ 15 days	N/U	Y		
Excluding other causes		N/U	Y	Score 1 if 3 tests of the following tests were performed and all were negative: HAV, HBV, HCV, Mycoplasma, Chlamydia, ANA, blood culture

ANA: anti-nuclear antibody; BSA: body surface area; HAV: hepatitis A virus; HBV: hepatitis B virus; HCV: hepatitis C virus; N: no; U: unknown; WBC: white blood cell; Y: yes.

**Table 3:** Associations between HLA alleles and DRESS syndrome

Drug	Associated HLA alleles	Population
Allopurinol	B*58:01	Han Chinese [27], European [87,88], Thai [89], Korean [90]
Carbamazepine	A*31:01	Han Chinese [91], European [92], Japanese [93]
	A*11, B*51	Japanese [93]
Dapsone	B*13:01	Han Chinese [94]
Nevirapine	DRB1*01:01, DRB1*01:02	African, Asian, European [95]
	Cw*4	African, Asian, European [95,96,97]
	B*35	Asian [95,98]
Phenytoin	B*13:01 and B*51:01	Han Chinese [82]

## Abbreviations

ALT	Alanine aminotransferase
ALP	Alkaline phosphatase
CCR	C-C motif chemokine receptor
CMV	Cytomegalovirus
CXCL	C-X-C motif chemokine
CXCR	C-X-C motif chemokine receptor
CYP	Cytochrome P
DiHS	Drug-induced hypersensitivity syndrome
DM	Diabetes mellitus
DRESS	Drug reaction with eosinophilia and systemic symptoms
EBV	Epstein-Barr Virus
FasL	Fatty acid synthase ligand
HHV	Human herpes virus
HLA	Human leukocyte antigen
HMGB-1	High mobility group box protein 1
IFN	Interferon
IL	Interleukin
IP-10	Interferon γ-induced protein-10
IVIG	Intravenous immunoglobulin
MHC	Major histocompatibility complex
MPE	Maculopapular exanthema
NSAID	Non-steroid anti-inflammatory drug
pDC	Plasmacytoid dendritic cell
SCAR	Severe cutaneous adverse reaction
SJS	Stevens-Johnson syndrome
TARC	Thymus activation-related chemokine
TCR	T cell receptor
TEN	Toxic epidermal necrolysis
T_EM_	Effector memory T cell
Th	Helper T cell
TNF	Tumor necrosis factor
Treg	Regulatory T cell
ULN	Upper limit of normal range
